# Metabolome fingerprinting reveals the presence of multiple nitrification inhibitors in biomass and root exudates of *Thinopyrum intermedium*

**DOI:** 10.1002/pei3.70012

**Published:** 2024-09-27

**Authors:** Sulemana Issifu, Prashamsha Acharya, Jochen Schöne, Jasmeet Kaur‐Bhambra, Cecile Gubry‐Rangin, Frank Rasche

**Affiliations:** ^1^ Institute of Agricultural Sciences in the Tropics (Hans‐Ruthenberg‐Institute) University of Hohenheim Stuttgart Germany; ^2^ Institute of Phytomedicine University of Hohenheim Stuttgart Germany; ^3^ School of Biological Sciences, Cruickshank Building University of Aberdeen Aberdeen UK; ^4^ Present address: Department of Plant and Environmental Sciences University of Copenhagen Frederiksberg C Denmark; ^5^ Present address: International Institute of Tropical Agriculture Nairobi Kenya

**Keywords:** ammonia oxidizing bacteria and archaea, BNI, Kernza®, metabolome, nitrification

## Abstract

Biological Nitrification Inhibition (BNI) encompasses primarily NH_4_^+^‐induced release of secondary metabolites to impede the rhizospheric nitrifying microbes from performing nitrification. The intermediate wheatgrass *Thinopyrum intermedium* (Kernza®) is known for exuding several nitrification inhibition traits, but its BNI potential has not yet been identified. We hypothesized Kernza® to evince BNI potential through the presence and release of multiple BNI metabolites. The presence of BNI metabolites in the biomass of Kernza® and annual winter wheat (*Triticum aestivum*) and in the root exudates of hydroponically grown Kernza®, were fingerprinted using HPLC‐DAD and GC–MS/MS analyses. Growth bioassays involving ammonia‐oxidizing bacteria (AOB) and archaea (AOA) strains were conducted to assess the influence of the crude root metabolome of Kernza® and selected metabolites on nitrification. In most instances, significant concentrations of various metabolites with BNI potential were observed in the leaf and root biomass of Kernza® compared to annual winter wheat. Furthermore, NH_4_^+^ nutrition triggered the exudation of various phenolic BNI metabolites. Crude root exudates of Kernza® inhibited multiple AOB strains and completely inhibited *N*. *viennensis*. Vanillic acid, caffeic acid, vanillin, and phenylalanine suppressed the growth of all AOB and AOA strains tested, and reduced soil nitrification, while syringic acid and 2,6‐dihydroxybenzoic acid were ineffective. We demonstrated the considerable role of the Kernza® metabolome in suppressing nitrification through active exudation of multiple nitrification inhibitors.

## 1 INTRODUCTION

Nitrogen (N) is the most required nutrient by crops relative to other elements (Shafreen et al., 2021). Despite it being so essential, N losses, through leaching, nitrous oxide (N_2_O) emissions, and surface run‐off, have deprived field crops of maximum utilization of N (Shafreen et al., 2021; Ussiri & Lal, 2013). This has led to complex environmental and climatic problems (Galloway et al., 2010; Ussiri & Lal, 2013), as well as increased economic costs to farmers (Smith et al., 2013). Nitrification, the microbially‐mediated oxidation of reduced N forms (e.g., ammonium [NH_4_^+^]) to nitrate [NO_3_^−^] is a key process that leads to these environmentally and economically relevant N losses (Deni & Penninckx, 1999; Mancinelli & Mckay, 1988; Prosser et al., 2020). Nitrification is mediated by ammonia‐oxidizing archaea (AOA) and bacteria (AOB), as well as complete ammonia oxidisers (comammox) in soils (Daims et al., 2015). Hence, controlling nitrification could serves as an effective means of mitigating N losses (Meng et al., 2021). This approach involves uncovering ecological alternatives to avoid the use of widely used synthetic nitrification inhibitors like dicyandiamide (DCD), 3,4‐dimethypyrazole phosphate (DMPP), and nitrapyrin (Konwar et al., 2016; Ussiri & Lal, 2013).

Sustainable management of N fertilizers to prevent N losses from agricultural land continues to engage the attention of practitioners (Shafreen et al., 2021). A central step towards this aim is to unravel the role of plants and their associated metabolome in controlling soil N fluxes (Nardi et al., 2020; Shafreen et al., 2021). Several decades ago, the tropical grass *Hyparrhenia filipendula* was found to inhibit nitrification (Munro, 1966). After this discovery, there have been a wealth of investigations into the role of root‐released phytochemicals of various plant species inhibiting nitrification. Phenolics, including tannins, caffeic acid, ferulic acid, *p* hydroxybenzoic acid, quercetin, isoquercitrin (Rice & Pancholy, 1972, 1973, 1974), vanillic acid (Kholdebarin & Oertli, 1994), coumarin (Hassanein et al., 2014), and vanillin (Egenolf, Schöne, et al., 2023), as well as other non‐phenolic metabolites (e.g., phenylalanine, linoleic acid) have been proven to have inhibitory potentials against AOB and AOA, and soil nitrification (Nardi et al., 2020). Such metabolites, with verified inhibition of nitrification, are collectively defined as putative nitrification inhibitors (Nardi et al., 2020). It is important to note that most of these putative metabolites, especially the phenolics, are ubiquitous in the ecosystem due to their multifunctional roles in the lifecycle of crops (Hoque et al., 2020; Min et al., 2015; Palanisamy et al., 2020), suggesting their presence in many crops.

Plant‐mediated nitrification inhibition, which involves the exudation of secondary metabolites elicited primarily by NH_4_^+^ to inhibit the rhizospheric nitrifiers from their nitrification activity, is called biological nitrification inhibition (BNI) (Subbarao, Wang, et al., 2007). Despite the important role of plants as mediators of BNI, some putative nitrification inhibitors like vanillic acid, ferulic acid, caffeic acid, *p* hydroxybenzoic acid, coumarin, and some other phytochemicals (Nardi et al., 2020; Rice & Pancholy, 1972, 1973, 1974), have not been tested *in planta* under different N types to verify their exudation and respective inhibition potential. BNI has been observed in several grasses, including maize (*Zea mays*) (Otaka et al., 2022), signalgrass (*Brachiaria humidicola*) (Egenolf et al., 2020; Gopalakrishnan et al., 2007; Subbarao et al., 2009), sorghum (*Sorghum bicolor*) (Subbarao, Nakahara, et al., 2013), rice (*Oryza sativa*) (Kaur‐Bhambra et al., 2023; Sun et al., 2016), wheat (*Triticum aestivum*; *Leymus racemosus*) (Bozal‐Leorri et al., 2022; Subbarao et al., 2021; Subbarao, Tomohiro, et al., 2007), and barley (*Hordeum vulgare*) (Fountain, 2022). It is considered to be an important nature‐based concept for regulating the N problems from agricultural fields (Subbarao et al., 2015). Among its attributes, BNI has been linked to significant reduction in N_2_O emissions (Galloway et al., 2010; Subbarao, Nakahara, et al., 2013), significant N legacy effect—benefiting succeeding crops and lowering N requirement in the ensuing years (Karwat et al., 2017), lower abundance of ammonia‐oxidisers carrying the ammonia oxidation genes (Sun et al., 2016; Tzanakakis & Paranychianakis, 2017), lower N leaching and surface run‐off (Sun et al., 2016), improved N uptake thereby increasing N use efficiency (NUE) (Bozal‐Leorri et al., 2022; Subbarao, Tomohiro, et al., 2007), and inhibition of growth of nitrifiers (Kaur‐Bhambra et al., 2022). In this regard, ongoing researches have identified a range of BNI‐positive plants and metabolites to enhance the sustainable management of N (Nardi et al., 2020). However, these efforts have been constricted to the identification of single and novel metabolites in each plant, despite the existence of multiple biological nitrification inhibitors in BNI‐positive plants (Egenolf et al., 2020; Gopalakrishnan et al., 2007;  Lu et al., 2022; Otaka et al., 2022), suggesting concurrent existence and exudation of these metabolites.

Most of the above‐mentioned attributes of BNI have also been observed in intermediate wheatgrass (*Thinopyrum intermedium*), a perennial cereal crop from which The Land Institute (Kansas, USA) bred the commercial cultivar Kernza®. Kernza® is thought to mimic natural grasslands in terms of relevant ecosystem functions and services (Audu et al., 2022; Crews, 2016; de Oliveira et al., 2020; Rasche et al., 2017). Accordingly, Kernza® has been designated a resource‐efficient crop (Duchene et al., 2020). For example, a higher NUE in Kernza® than annual wheat has been reported (Sprunger et al., 2018). Furthermore, N_2_O emissions and total N leaching from Kernza® fields were significantly lower compared to annual wheat (Daly et al., 2022; Culman et al., 2013). Additionally, N need and consequent uptake was also reduced by up to 50% in Kernza® during its second growing season (Fagnant et al., 2023), which underscores its residual N sequestration ability compared to annual wheat (Kell, 2011; Peixoto et al., 2022; Pierret et al., 2016). Recently, Audu et al. (2022) reported a lower abundance of bacterial ammonia oxidisers in Kernza® fields relative to annual wheat.

These results indicate a potential of Kernza® to inhibit nitrification, an eco‐physiological trait linked to the plant's perenniality and enduring root system (Sprunger et al., 2018). However, the critical role of the crop's metabolome has not been addressed in verifying the BNI‐like potential of Kernza®. Thus, we hypothesize that the presence and release of nitrification inhibitors in the tissue and root exudates of Kernza® reveal a considerable potential to mitigate nitrification. Consequently, we expect NH_4_^+^ nutrition to induce a higher exudation of nitrification inhibitors than NO_3_^−^ nutrition, thereby inhibiting the growth of ammonia‐oxidisers. To verify these assumptions, a metabolome fingerprinting approach was adopted to characterize the metabolome of Kernza® and to ascertain the influence of different N forms on the exudation of BNI metabolites in Kernza®. Concurrently, bioassays, using selected strains of ammonia‐oxidisers (AOB, AOA) were performed to assess the effect of crude root exudates of Kernza® on pure cultures of the ammonia‐oxidisers. These bioassays were complemented by selected phytochemicals to ascertain their effect on pure cultures of ammonia‐oxidisers and soil nitrification.

## 2 MATERIALS AND METHODS

### 2.1 Kernza® and annual winter wheat biomass sampling

Kernza® and annual winter wheat (*Triticum aestivum*) fields were established in 2017 and 2019, respectively, in Maubec (France, 45°34′5″ N, 5°15′58″ E). At the time of sampling in June 2021, the climatic conditions revealed a mean annual rainfall of 843 mm and an average temperature of 11.6°C. The soil had a loamy texture and a soil pH of 7.5, a C/N ratio of 19.4 and 14.2 for the Kernza® and annual winter wheat fields, respectively. The total N of the soils were 2.53 and 4.05 g kg^−1^, and the total organic C were 50 and 29 g kg^−1^ for the Kernza® and annual winter wheat fields, respectively. Plant tissues (leaves and roots from healthy plants) were randomly sampled from flowering plants (BBCH 61–65) to capture the heterogeneity of the fields. The plants were uprooted, and the below‐ground biomass was excised from the above‐ground biomass, each bagged separately and shipped on ice to the laboratory. On arrival, the root samples were washed under running deionized water to remove soil particles and kept at 4°C until further processing.

### 2.2 Metabolites extraction from biomass of Kernza® and winter annual wheat

The root and shoot samples were air‐dried in a ventilated dark room at about 25°C until dryness (about 10 days) and milled into fine particles. The used extraction method has been described previously (das Neves & Gaspar, 1990; Feitoza et al., 2020; Luthria et al., 2015; Mashabela et al., 2022), except here with a few modifications. Briefly, for the untargeted metabolomics, 2 g of dry matter weight (DMW) were dispensed into 15 mL falcon tubes containing 10 mL of 80% methanol, and then subjected to an ultrasonic bath (Elma Schmidbauer GmbH) for 30 min at 50°C. The samples were then centrifuged at 2500 rpm for 5 min under room temperature, the supernatant (approximately 8 mL) was filtered (0.22 μm PVDF syringe filter) and stored at −20°C for downstream GC–MS/MS analysis. For the targeted metabolomics, 2 g DMW were dispensed in 15 mL falcon tubes containing 10 mL of 80% methanol, and then subjected to an ultrasonic bath for 60 min at 25°C. The samples were then centrifuged at 2500 rpm for 15 min under room temperature, the supernatant (approx. 8 mL) was filtered (0.45 μm PVDF syringe filter), concentrated under a gentle stream of nitrogen gas to a 2 mL final volume, and stored at −20°C for downstream HPLC‐DAD/MS analysis.

### 2.3 Kernza® growth and root exudates collection

Kernza® seeds from ISARA‐Lyon (France) were sterilized (Data S1), plated on moist papers, and kept in a dark incubator at 25°C until sprouting. The seedlings were transplanted into sandy soil in a germination tray, with five seedlings per spot and placed in a growth chamber with a 14 h day length. The growth chamber hat a light intensity of 525 W m^−2^, air humidity was maintained at 70%, and day and night temperatures were set at 28 and15°C, respectively. Plants were supplied with half‐strength nutrient solution every 2 days for a period of 23 days. The plants were then transferred into 20 L hydroponic growth boxes filled with full‐strength solution, each box containing 30 plants held in place with sponges (Data S2). The full strength nutrient solution which we optimized for the first time for hydroponic growth of Kernza® (concentrations given in μM) contained NH_4_NO_3_ (4000), K_2_HPO_4_ (1500), MgSO_4_*7H_2_O (2100), CaCl_2_*2H_2_O (3350), KNO_3_ (4500), Ca(NO_3_)_2_*4H_2_O (4000), H_3_BO_3_ (4.63), MnSO_4_*H_2_O (9.2), ZnSO_4_*7H_2_O (1.7), CuSO_4_*5H_2_O (0.596), Na_2_MoO_4_*2H_2_O (0.22) and Fe‐EDTA (21.6). The pH was adjusted with HCl and Na_2_CO_3_ up to pH 6.0. The nutrient solution was replaced every third day. Aeration of the system was achieved with a vacuum pump supplying air intermittently at 15‐min intervals.

A preculture treatment similar to Egenolf et al. (2021) was instituted, whereby when the plants were 23 days old, the crops were divided into two sets and provided with either NH_4_^+^ or NO_3_^−^ as the sole N source, which was achieved through the equivalent addition of (NH_4_)_2_SO_4_/NH_4_Cl or KNO_3_ in place of NH_4_NO_3_, Ca(NO_3_)_2_*4H_2_O, and KNO_3_, respectively. Root exudates were collected at 30 and 45 days after transfer (DAT) using a randomized collection setup with four and three replicates at 30 and 45 DAT, respectively. Each replicate consisted of four plants whose roots were gently immersed into one‐liter glass boxes covered with aluminium foil, filled with one‐liter of the corresponding trap solution (containing NH_4_^+^ or NO_3_^−^ as N source), and supplied with bubbles of air produced by a vacuum pump. The root exudates were collected for approximately 18 h at 30 DAT, and the plants were then returned to their respective hydroponic growth tank until 45 DAT, when exudates were collected again for about 6 h. Collection of exudates began at 9:00 am, that is 3 h after onset of irradiation.

After collecting crude exudates, the trap solution was filtered through Whatman Grade 2 to remove debris. One hundred mL filtrate of each replicate was aliquoted for use in the bioassay and total amino acids analysis. The filtrate was subjected to liquid phase extraction to extract apolar metabolites (Egenolf et al., 2021).

### 2.4 GC–MS/MS analyses

Extracted samples were dried under a gentle steam of nitrogen gas and reconstituted in 100 μL acetonitrile and 100 μL N,O‐Bis(trimethylsilyl)trifluoroacetamide (BSFTA). The vial with the reconstituted samples was closed and shaken at 300 rpm, 60°C for 30 min (Eppendorf ThermoMixer® C) before GC–MS/MS measurements. Fingerprinting and identification of metabolites were carried on a GC (Agilent 7890B) coupled with an Autosampler (Agilent 7693A) and an MS (Agilent 7000D Mass spectrometer Triple‐Quadrupole). The set‐up of the GC was as follows: an Agilent HP‐5MS UI (30 m × 250 μm × 0.25 μm) was used for metabolite separation, and the heating of samples and stationary phase in the column was achieved with an oven conditioned as 80°C (2 min) −5°C/min −325°C (2 min) and an injector temperature of 250°C. One μL of the derivatised sample was injected with a pulsed splitless technique. Temperature for the transfer line was set at 250°C at a flow rate of 1 mL min^−1^, using Helium as a carrier gas to achieve a high separation efficiency. For MS, at the source, electron ionization was conducted at 230°C, 70 eV with a quadrupole temperature of 150°C, and sample analysis was done with an MS1‐scan range at m/z 50–600. The MassHunter Workstation (Agilent) GC/MS Data Acquisition (Version 10, 2019) software was used for data acquisition and analysis. The metabolites identified through GC–MS/MS were classified as putative metabolites, relying on published BNI identifications (Egenolf, Schöne, et al., 2023; Hassanein et al., 2014; Kholdebarin & Oertli, 1994; Lu et al., 2022; Nardi et al., 2020; Wang et al., 2023), or candidate metabolites (those annotated for testing) based on a combined literature and chemical database search (https://www.reaxys.com/).

### 2.5 HPLC‐DAD/MS

Instrumentation, standard preparation, and calibration as detailed in Data S3 as well as measurements followed existing protocols (Egenolf, Schöne, et al., 2023; Were et al., 2022). The details for the elution gradient are provided in Data S4.

### 2.6 Growth bioassays

#### 2.6.1 Preparation of crude root exudates and metabolites

For the crude root exudates used in the growth inhibition bioassay, 100 mL aliquots from Kernza® root exudates (seven replicates) were extracted in the same way as the extractions for the metabolome fingerprinting with a few modifications. The extracted root exudates were fully dried under a vacuum evaporator for an hour to remove all remnants of ethyl acetate, and the dried extracts were redissolved in 50 μL of 100% DMSO, pooled to yield 350 μL, and stored at −20°C until use. Five commercial HPLC‐grade compounds 4‐hydroxy‐3,5‐dimethoxybenzoic acid (syringic acid), 3‐(3,4‐dihydroxyphenyl)‐2‐propenoic acid (caffeic acid), 4‐hydroxy‐3‐methoxybenzaldehyde (vanillin), 4‐hydroxy‐3‐methoxybenzoic acid (vanillic acid), 2,6‐dihydroxybenzoic acid, and methyl 4‐hydroxy‐3,5‐dimethoxybenzoate (methyl syringate) were also bought from Thermo Fisher Scientific (China and UK). These metabolites were dissolved in 100% DMSO and serially diluted up to 2000 μM in DMSO.

#### 2.6.2 Cultivation of nitrifiers and supplementation with crude root exudates and metabolites

Four AOB (*Nitrosomonas europaea* ATCC 19718, *Nitrosospira multiformis* ATCC 25196, *Nitrosospira tenuis* NV12 (Harms et al., 1976), *Nitrosospira briensis* 128 (Rice et al., 2016)) and one AOA (*Nitrososphaera viennensis* (Tourna et al., 2011)) pure cultures were used for the growth bioassays. AOB were cultivated in the Skinner & Walker freshwater media (Skinner & Walker, 1961) with modifications, using phenol red as pH indicator and 1 M Na_2_CO_3_ for adjustment of pH, while the AOA was cultivated in modified freshwater media (Tourna et al., 2011). The growth assays were performed using 1% nitrifier inoculum in 10 mL growth media (in 30 mL universal plastic bottles) with cultures incubating at 28°C and 35°C for AOB and AOA, respectively. Microbial treated cultures were supplemented with 10 μL of the crude root exudate or authentic metabolites, while the controls were supplemented with 10 μL of 100% DMSO, which resulted in 0.1% (v/v) DMSO in all the cultures. As compound supplementation could modify the medium pH (Data S5), the pH of the controls was initially adjusted to those of the treatments (~ pH 7.5) with 10% (v/v) HCl and 5% (v/v) Na_2_CO_3_ for the AOB, while the pH for AOA media remained unchanged. All assays were set up in quadruplicates. AOB and AOA growth was monitored using NO_2_^−^ accumulation over time as proxy, and growth inhibition was estimated as previously described (Kaur‐Bhambra et al., 2022). NO_2_^−^ concentration was determined using the Griess test (Shinn, 1941).

### 2.7 Effect of metabolites on soil nitrification

Metabolites showing some microbial inhibition in the growth bioassays were progressed for further soil testing, using a previously published procedure (Egenolf, Schad, et al., 2023; Tzanakakis & Paranychianakis, 2017; Zhao et al., 2020) with a few modifications. Briefly, a Luvisol soil with texture silt loam (3.4% sand, 76.2% silt, 20.5% clay), a pH of 6.8, a total carbon and nitrogen of 1.11% and 0.09%, respectively (C/N ratio: 12) and a C_org_ of 1.03% was collected from Meiereihof, an experimental site of the University of Hohenheim. The soil was air‐dried and passed through a 2‐mm sieve before use. Ten gram of the soil was weighed into 40 mL vials and supplied with vanillic acid (Sigma Aldrich, Darmstadt, Germany) at 1000 μg g^−1^ of dry‐weight soil. Each vial was provided with 1 mL of deionized water initially. Afterwards, NH_4_(SO_4_)_2_ was supplied to the vials, resulting in 182 N mg kg^−1^, and sealed with a parafilm perforated with a pin on the top to allow aeration during the experimental period. Two‐hundred μL of distilled water was supplied to the vials every 9 days to achieve and maintain a 60% water‐filled pore space (WFPS). A control treatment without vanillic acid addition was established in parallel. All treatments were incubated at 28°C in the dark for 18 days. NH_4_^+^ and NO_3_^−^ were extracted from samples destructively (10 g soil) on days 0, 4, 7, 11, 14, and 18 with 100 mL of 2 M KCl (1:10) after shaking the soil solution for 30 min on a shaker. The soil solution was filtered through Whatman No. 1 filter paper and the filtrate was quantified colorimetrically for NH_4_^+^ as green ammonium salicylate complex at 667 nm and NO_3_^−^ as yellow nitro‐salicylate at 410 nm. Nitrification was estimated as the accumulation of total NO_3_^−^‐N per timepoint.

### 2.8 Statistics

Statistical analyses for metabolome fingerprinting and quantitation in the plant tissues as well was done with JMP Pro 17 software, while GraphPad Prism 10.0.2 was used for visualization. Remaining tests (hydroponics, soil incubations, and bioassays) were analyzed with R v.4.3.1 using the package tidyverse and multcom while figures and plots were done with ggplot2. All data were subjected to outlier checks within treatment replicates using the univariate and multivariate commands of JMP Pro 17 with the packages robust fit outliers (r FO) with a K sigma 4 and robust principal components analysis (r PCA) with Lambda 0.535. Data homoscedasticity and normality of residuals were assessed by a graphical screening of the residuals and Q‐Q plots. After satisfying the parametric tests linear models were fitted. For the metabolome fingerprinting and quantitation in the plant tissues, crop type was fitted as fixed effects and metabolites as response variables. In the hydroponics experiment to test the effect of NH_4_^+^ and NO_3_^−^ as sole N source on metabolite exudation, the differences in concentration of metabolites were linearly modeled for the interactive interactions of timepoint and N source, for each metabolite separately. While for the bioassays, changes in strain inhibition due to the metabolite type and concentration were for each ammonia‐oxidiser separately modeled. The effect of vanillic acid on soil nitrification was analyzed by fitting its concentrations as fixed effects and NH_4_^+^ and NO_3_^−^ concentrations as response variables for each timepoint. Test for statistical differences between treatments was accomplished by ANOVA, followed by Tukey post hoc analyses.

## 3 RESULTS

### 3.1 Untargeted identification of putative and novel putative metabolites in Kernza® biomass

The untargeted and qualitative screening was done to identify the full range of phytochemicals (either putative or candidate metabolites) present in the biomass of Kernza®. A broad range of putative phenolics and non‐phenolic metabolites, including amino acids, were identified in both Kernza® and winter wheat biomass (Data S6—Table). The presence of metabolites differed between the roots and leaves of both plants. In total, 8 and 11 putative BNI metabolites were annotated in the leaves and roots of Kernza®, respectively, whereas 7 and 9 metabolites were identified in wheat leaves and roots, respectively.

### 3.2 Targeted identification and quantification of putative metabolites

**FIGURE 1:**
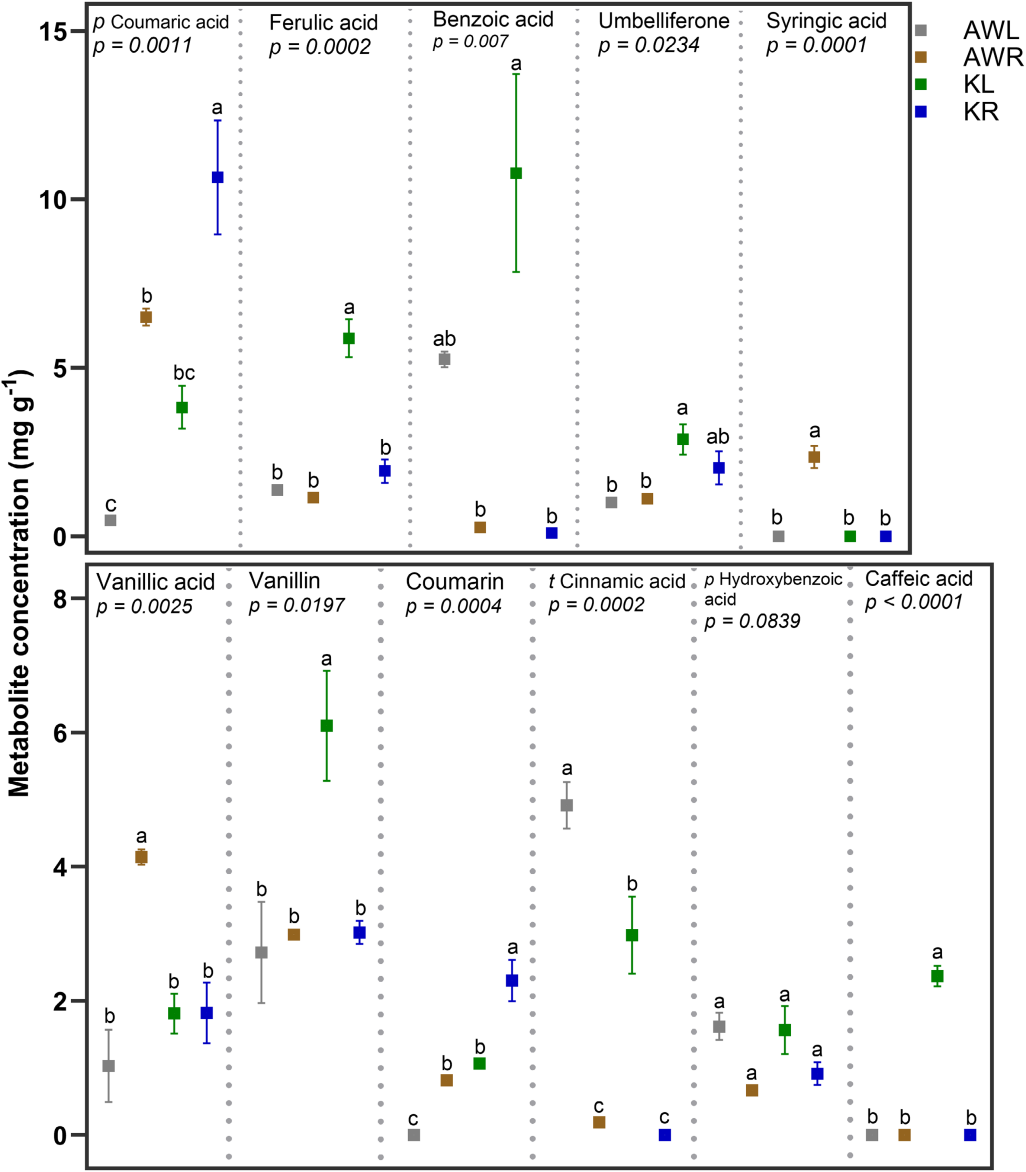
Concentrations of putative metabolites in root and leaf tissues. Concentrations are presented as least square means of three replicates with standard error for each treatment. AWL, annual winter wheat leaves; AWR, annual winter wheat roots, KL, Kernza® leaves; KR, Kernza® roots. Within each graph, the *p*‐value indicates the ANOVA statistical significance and different letters indicate significant differences following a Tukey post hoc test.

Targeted identification and quantification of metabolites were done to validate the qualitative identification of putative and candidate BNI metabolites and to compare the concentrations of these metabolites in Kernza® and annual winter wheat biomass. In total, 11 phenolics were identified and quantified accordingly in different parts of the plants (Figure 1). Concentrations of the metabolites depended on the plant tissue (root or leaf) and the plant species. Specifically, higher quantities of putative metabolites, namely ferulic acid, *p* coumaric acid, coumarin, and benzoic acid were found in Kernza® roots or leaves compared to annual winter wheat biomass. Syringic acid and caffeic acid were found exclusively in annual winter wheat roots and Kernza® leaves, respectively. Concentrations of other phenolics like vanillin and umbelliferone were highest in Kernza® leaves and roots. Vanillic acid revealed highest concentrations in annual wheat roots. Cinnamic acid was highest in annual winter wheat leaves, followed by Kernza® leaves. *p* hydroxybenzoic acid had no significant differences in concentrations in both plants and tissues. Finally, protocatechuic acid was not identified in both crops, although it was found in the earlier qualitative fingerprinting (Data S6—Table).

### 3.3 Assessment of N forms (NH_4_^+^ vs. NO_3_^−^) on the exudation of putative metabolites

**FIGURE 2:**
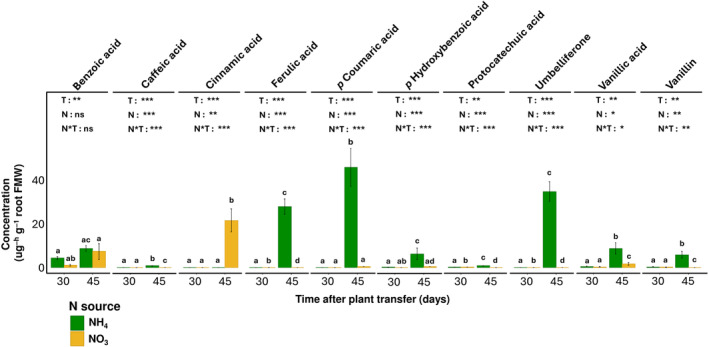
Effect of different N sources (NO_3_^−^ and NH_4_^+^) and crop age on the root exudation rate of bioactive phenolics with BNI effects. The concentration of all phenolic compounds was indicated per unit of root fresh matter weight (FMW). Data is presented as least square means with standard error for four and three replicates at time points 30 DAT and 45 DAT, respectively. Different letters represent significant differences between N sources within and across time points for each metabolite within each subplot. For each metabolite, the effect of time after transplantation (T), N sources (N) and their interaction (N*T) was analyzed using ANOVA where the where ns, *, **, and *** represent the significance of the effect at *p* > .5, *p <* .01, *p <* and *p <* .001, respectively.

Five metabolites (benzoic acid, vanillin, protocatechuic acid, vanillic acid and *p* hydroxybenzoic acid) were detected at the two tested time points (30 and 45 DAT), while caffeic acid, ferulic acid, *p* coumaric acid, and umbelliferone, were exclusively released at 45 DAT (Figure 2). Comparatively, under both NO_3_^−^ and NH_4_^+^ nutrition, metabolites were significantly higher at 45 DAT than 30 DAT. In addition, at 45 DAT, the NH_4_^+^ rather than NO_3_^−^ nutrition, triggered the exclusive exudation of caffeic acid, ferulic acid, protocatechuic acid, vanillin, and umbelliferone, while it stimulated a significantly higher exudation of *p* coumaric acid (*p* = 8.9 × 10^−5^), vanillic acid (*p* = .012), and benzoic acid (*p* = .008). Phenylalanine was also found in the root exudates when amino acids were quantified (data not shown).

### 3.4 Microbial growth inhibition by selected metabolites and crude root exudates of Kernza®

**FIGURE 3:**
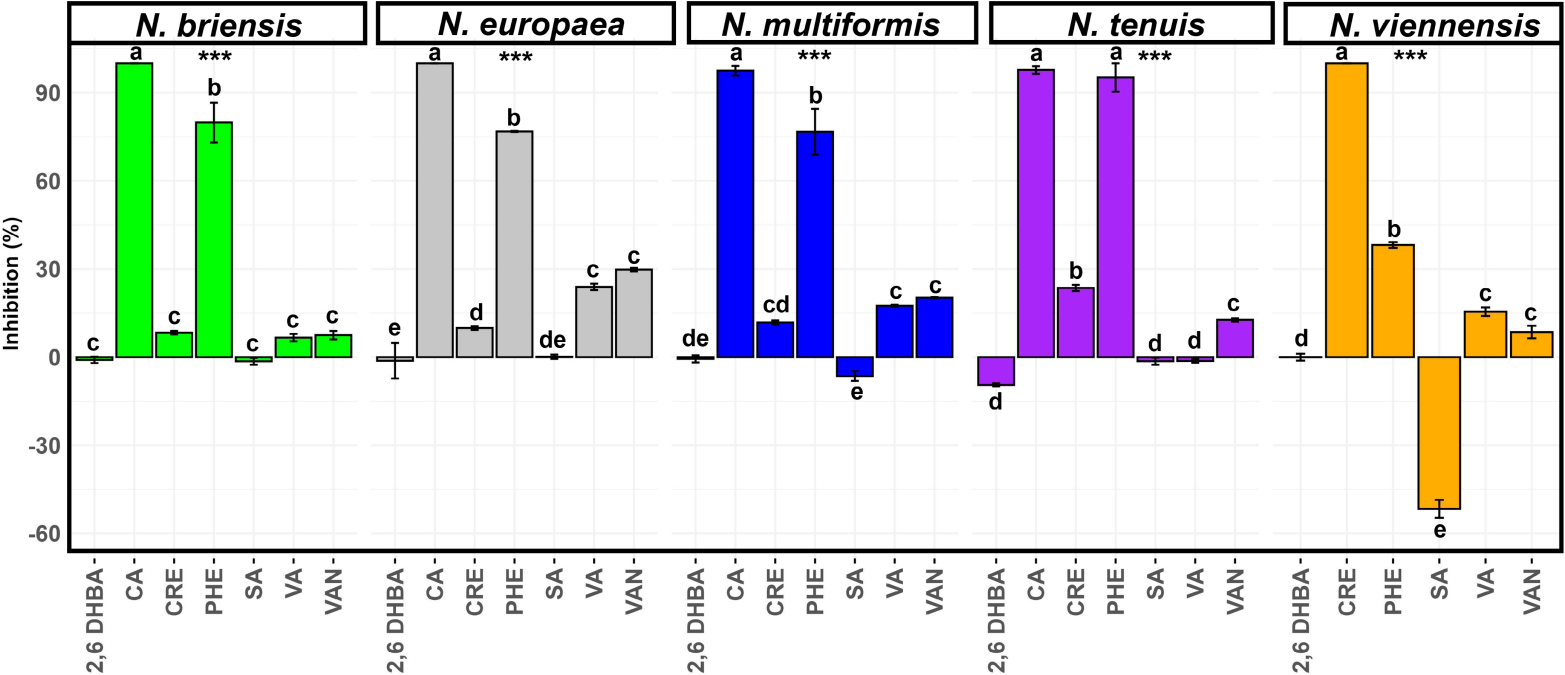
Effect of selected metabolites and crude root exudate of Kernza® on the growth of nitrifying microbes. Results represent least means square (±SE) for four replicates. Inhibitions are relative to the controls. Within each sub‐plot, different letters above each bar indicate significant differences between treatments (metabolites) on microbes, while the asterisks *** represents significant different between treatments (*p* < .0001). Metabolites 2,6 DHBA, 2,6 dihydroxybenzoic acid, CA, caffeic acid, PHE, phenylalanine, SA, syringic acid, VA, vanillic acid, VAN, vanillin, and CRE, crude root exudates. Metabolites were tested at 500 and 50 μM for the AOB and AOA, respectively.

We tested the inhibitory potential of the crude root exudates of Kernza® as well as several phenolics; 4‐hydroxy‐3,5‐dimethoxybenzoic acid (syringic acid), 3‐(3,4‐dihydroxyphenyl)‐2‐propenoic acid (caffeic acid), 4‐hydroxy‐3‐methoxybenzaldehyde (vanillin), 4‐hydroxy‐3‐methoxybenzoic acid (vanillic acid), 2,6 dihydroxybenzoic acid, an amino acid phenylalanine (Figure 3), and methyl 4‐hydroxy‐3,5‐dimethoxybenzoate (methyl syringate), which were selected based on the metabolome fingerprinting and derivational relationship with putative metabolites on multiple pure cultures of AOB and AOA (Data S7). The inhibitory effect of the crude root exudates and the metabolites was significantly (*p* < 0.0001) variable among the test strains. Crude root exudates inhibited the AOB growth up to 24%, and completely inhibited the AOA growth. Caffeic acid the most effective inhibitor among the metabolites tested—inhibiting all strains up to 100%. Phenylalanine achieved 38% (*N*. *viennensis*) and between 76% (*N*. *multiformis* and *N*. *briensis*) and 96% (*N*. *europaea* and *N*. *tenuis*) inhibition of the AOB. Vanillic acid inhibited three AOB growth between 7% and 24%, with minimal effect on *N*. *tenuis* (−1.3%). It inhibited AOA (*N*. *viennensis*) (15%) growth at a much lower concentration (50 μM). In contrast, 2,6‐DHBA and methyl syringate (tested on only two strains—*N*. *europaea* and *N*. *multiformis*—Data S7) showed contrasting effects with no inhibition to mild stimulatory effects on different tested AOB and AOA strains, with no noticeable inhibition even when tested at 1000 μM. Also, syringic acid was found not to have any inhibitory effect against any of the AOB strains even when tested at 800 μM—rather stimulating *N*. *viennensis* up to 52%.

### 3.5 Effect of metabolites on soil nitrification

**FIGURE 4:**
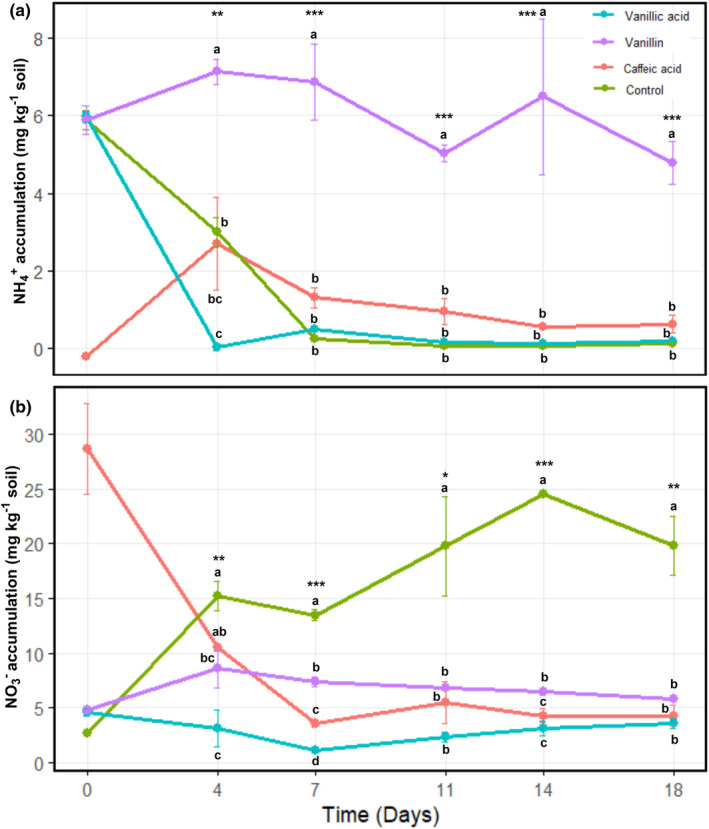
Effect of metabolites on (a) exchangeable NH_4_^+^‐N content (*y*‐axis) over time in days (*x*‐axis); (b) potential nitrification activity expressed as total mineral nitrogen NO_3_^−^‐N produced (y‐axis) over time in days (*x*‐axis). Results represent least square means (±SE) for three replicates. Different letters on the bars indicate significant differences (***, **, *p < .001, .01, .05, respectively) between treatments and control for each under each timepoint.

Three metabolites (caffeic, vanillic acid, and vanillin) were tested for their effect on soil nitrification. Nitrification was inhibited in all soils amended with the metabolites relative to the unamended soil (control) (Figure 4a). Compared to the unamended control (*n* = 5, timepoints from day 4) which accumulated an average NO_3_^−^ concentration of 19 (SE ± 1.8) mg kg^−1^ soils amended with caffeic acid, vanillic acid, and vanillin achieved average NO_3_^−^ concentrations of 6 (SE ± 2.3), 3 (SE ± 0.4), and 7 (SE ± 0.4) mg kg^−1^, respectively. There was a significant (*p* < .0001) difference between the unamended soil and amended soils at all timepoints beginning from day 4, while significant differences between the amended soils was recorded for some timepoints. Correspondingly, NH_4_^+^ accumulation took a declining trend over the period, except for vanillin where a relative stability was noticed (Figure 4b). Average NH_4_^+^ accumulation in the unamended soil was 0.70 (SE ± 0.5) mg kg^−1^ while an average of 1.2 (SE ± 0.3), 0.2 (SE ± 0.3), and 6.1 (SE ± 0.4) were recorded for caffeic acid, vanillic acid, and vanillin, respectively. Differences in NH_4_^+^ accumulation between the unamended and amended soils were significant (*p* < .0001) at all timepoints, whereas there were no significant differences between the amended soils, except for day 4. Overall, vanillic acid achieved the highest suppression of NO_3_^−^ accumulation, while vanillin curiously maintained a stable concentration of NH_4_^+^.

## 4 DISCUSSION

We demonstrated the mediating role of Kernza® metabolome in the suppression of nitrification by showing that NH_4_^+^, rather than NO_3_^−^, induces significant, and in some cases, exclusive exudation of multiple nitrification inhibitors, whereas previously, plant‐mediated control of nitrification has been assessed through the identification of single and novel metabolites (Lu et al., 2022; Nardi et al., 2020), we have presented evidence of the co‐exudation of putative metabolites in Kernza®. This is the first time a gamut of putative metabolites has been assessed in vivo under contrasting N sources. Kobayashi et al. (1996) and Zhang et al. (2020) have documented the co‐exudation and co‐existence of similar bioactive metabolites (i.e., *p* coumaric acid, cinnamic acid, *p* hydroxybenzoic acid, vanillic acid, ferulic acid) in the rhizosphere and root exudates of wheat, maize, and soybean in varying amounts. The inducement of exudation of nitrification inhibitors by NH_4_^+^, as found in Kernza®, is a plant mechanism to control nitrification (Nardi et al., 2020), which has been reported in grasses like *Brachiaria humidicola* (Egenolf et al., 2020; Gopalakrishnan et al., 2007; Subbarao et al., 2009), rice (Kaur‐Bhambra et al., 2023; Lu et al., 2022; Sun et al., 2016), maize (Otaka et al., 2022), and wheat. Thus, the exudation of bioactive metabolites functioning as nitrification inhibitors by plants has been noted as an important ecosystem service and nature‐based solution to control nitrification (Subbarao, Nakahara, et al., 2013). Consequently, we argue that, attributes which signify controlled nitrification by plants (Subbarao, Rao, et al., 2013; Sun et al., 2016), as reported in Kernza®, including low N_2_O emissions, reduced N leaching (Daly et al., 2022; Pugliese et al., 2019), nitrogen use efficiency (Sprunger et al., 2018), lower bacterial *amoA* abundance (Audu et al., 2022), among others, are linked to the presence of nitrification inhibitors in the root exudates and biomass of Kernza®.

Aside the active exudation of nitrification inhibitors, we found evidence of the existence of multiple nitrification inhibitors in the biomass of Kernza® and annual wheat, a finding in line with earlier reports on *Suaeda salsa* (Wang et al., 2023), *Brachiaria* (Gopalakrishnan et al., 2007), and wheat (Luthria et al., 2015). These inhibitors could be released during turnover through decomposition, resulting in—the suppression of nitrification (Gopalakrishnan et al., 2007) and—high legacy nitrogen as found with *Brassicaceae* tissues (Brown & Morra, 2009) and *Brachiaria* (Nakamura et al., 2020). Indeed, such turnover effect may also explain the high legacy N observed in Kernza® fields (Daly et al., 2022; Sprunger et al., 2018). A similar observation has been reported by Karwat et al. (2017), who reported a strong legacy N of a *Brachiaria* spp. pasture in succeeding maize. Consequently, mediation of nitrification by Kernza® could be exerted through the active release of multiple nitrification inhibitors or passively during turnover.

Most importantly, we confirmed the active suppression of nitrifiers by Kernza® through antimicrobial tests of its root exudates, which suppressed the growth of all test strains. This is the first evidence for the antimicrobial property of root exudates of Kernza® against multiple nitrifier strains (AOB and AOA). The ability of crude root exudates of plants to suppress nitrifiers is an important approach to confirm a plant's ability to inhibit nitrification as reported in sorghum (Li et al., 2021; Subbarao, Nakahara, et al., 2013), rice (Kaur‐Bhambra et al., 2023), and *Brachiaria* (Subbarao et al., 2006). Additionally, we verified the inhibitory potential of the detected and characterized putative metabolites. Antimicrobial tests of four isoforms of hydroxybenzoic acid (i.e., vanillic acid, 2,6 dihydroxybenzoic acid, syringic acid, vanillin) revealed significant inhibitory effect of vanillic acid and vanillin on multiple nitrifier strains. However, contrary to previous reports (Lu et al., 2022), no effect was revealed for syringic acid and 2,6 dihydroxybenzoic acid. Vanillic acid and vanillin are known to inhibit the growth of nitrifiers and soil nitrification (Egenolf, Schad, et al., 2023; Kholdebarin & Oertli, 1994). Caffeic acid was also confirmed as a nitrification inhibitor in line with previous reports (Kolovou et al., 2023). It completely inhibited all test strains and achieved significant inhibition of soil nitrification (Rice & Pancholy, 1974). Additionally, phenylalanine, a previously characterized nitrification inhibitor (Nardi et al., 2020; Tzanakakis & Paranychianakis, 2017), which we detected in the root exudates of Kernza®, strongly inhibited all test strains.

We assume that the mode of action of vanillic acid and vanillin against nitrifiers is through the disruption of their cell walls, making them permeable. This is because vanillic acid is capable of decreasing intracellular ATP and pH, ultimately leading to cell death (Maisch et al., 2022). It is also possible that the ammonia monooxygenase (AMO) could be blocked by vanillin and vanillic acid, considering that both metabolites share chemical properties with syringic acid which has been reported to exhibit that kind of mode of action (Lu et al., 2022). Further, vanillic acid was shown to lower N mineralization by enhancing immobilization of N through microbial activity stimulation, thereby leading to lower N_2_O emissions (Frimpong et al., 2014). Other possibilities regarding lower NO_3_^−^‐N accumulation include the incorporation of NH_4_^+^ into vanillic acid through a chemical reaction under continuous aeration (Flaig & Schulze, 1952; Kholdebarin & Oertli, 1994) and stimulation of immobilization of NH_4_^+^ by heterotrophic microbes (Egenolf, Schad, et al., 2023; Karwat et al., 2017).

Further investigations are needed to establish the exact mode of action of vanillic acid, caffeic acid, and vanillin as nitrification inhibitors. The mode of action for caffeic acid against nitrifiers has not been investigated, but its antimicrobial effect against *Staphylococcus aureus* and *Escherichia coli* has been reported (Andrade et al., 2015). Regarding 2,6‐dihydroxybenzoic acid, which exhibited stimulatory behavior on nitrifiers, it was found to inhibit *E*. *coli*, *B*. *subtilis*, and *S*. *enteritidis* (Kalinowska et al., 2021). These contrasting findings suggest that not all metabolites with antimicrobial effects may necessarily be characterized as nitrification inhibitors—thus reinforcing the divergent effects of isoforms of bioactive metabolites (Egenolf et al., 2020; Kalinowska et al., 2021). In general, phenolics, including vanillic acid, vanillin, and caffeic acid, exhibit a potential to disrupt cytoplasmic membranes which constitute their mode of action against microbes (Andrade et al., 2015)—possibly including nitrifiers.

Lastly, it is important to also underscore that some nitrification inhibitors function effectively or ineffectively depending on the pH of the environment (Lu et al., 2022). Even though we took steps to ameliorate any confounding pH effect, future studies should consider testing the metabolites under multiple pH conditions.

## 5 CONCLUSION AND OUTLOOK

This study explored the intricate role of the metabolome of *Thinopyrum intermedium* (Kernza®) to inhibit nitrification, focusing on diverse bioactive phytochemicals. The biomass of Kernza® and annual winter wheat contained multiple BNI metabolites, with Kernza® exhibiting significantly higher concentrations of most of the metabolites. Kernza® exuded multiple nitrification inhibitors in significant concentrations, and in some instances, exclusively under NH_4_^+^ rather than NO_3_^−^. Kernza® root exudates effectively inhibited test strains of AOB and AOA in pure culture assays. Vanillic acid, caffeic acid, vanillin, and phenylalanine, which were found in both the biomass and root exudates of Kernza®, displayed antimicrobial abilities against multiple strains of AOB and AOA, along with a significant reduction in soil NO_3_^−^ accumulation. Our findings underscore the importance of reevaluating the collective role of the metabolome of plants, encompassing a wide array of phytochemicals in achieving nitrification inhibition. Given the concurrent exudation of multiple putative BNI metabolites, future research should delve into evaluating the BNI potential of Kernza® focusing on finding which metabolite(s) mediate BNI in Kernza®. Furthermore, the discovery of multiple nitrification inhibitors concurrently exuded in response to NH_4_^+^ necessitates an investigation into how these phytochemicals coexist and interact to affect nitrification. It is essential to verify whether these inhibitors act synergistically to enhance nitrification inhibition. In conclusion, the metabolome of Kernza® contains a considerable presence of putative nitrification inhibitors, which altogether, may mediate nitrification inhibition.

## FUNDING INFORMATION

Anton & Petra Ehrmann‐Foundation; BiodivClim ERA‐Net COFUND programme and German Research Foundation (grant RA 1717/8–1); Royal Society University Research Fellowship (URF150571).

## CONFLICT OF INTEREST STATEMENT

The authors declare no conflict of interests.

## Supporting information


Data S1.

Data S2.

Data S3.

Data S4.

Data S5.

Data S6.

Data S7.

## Data Availability

The data that support the findings of this study are available on request from the corresponding author. The data are not publicly available due to privacy or ethical restrictions.
