# Supplementary material for: Metabolome fingerprinting reveals the presence of multiple nitrification inhibitors in biomass and root exudates of Thinopyrum intermedium
Source: Plant Environ Interact. 2024 Sep 27;5(5):e70012. doi: 10.1002/pei3.70012 (PMC11431351; doi:10.1002/pei3.70012)
Supplement: Supplementary file 1 — Data S1. [file PEI3-5-e70012-s004.pdf]

Prepare: 70% EtOH

50% bleach

sterile dH2O

sterile tips (1ml and/or 200 [illegible]l)

a timer

- Put your seeds in a 1.7 ml tube or a falcon tube (depends on the amount of your seeds). Add enough 70% EtOH. Suspend the seeds in EtOH by turning the tube for 2 min.
- Pour out the EtOH. Add enough volume of 50% bleach. Do the bleach treatment for 5-10 min. Resuspend the seeds from time to time.
- Discard the bleach.
- Wash seeds thoroughly with a large amount of sterile dH2O.
- Discard the water. Repeat step 3 for 3 more times.
- Your seeds are ready for use.

Note: a. Don’t leave seeds in EtOH for too long. It may kill the seeds.

b. Wash off the bleach as completely as possible. Bleach could be toxic to seedlings.

c. Do step 4-6 in a sterile hood!
