# Supplementary material for: Metabolome fingerprinting reveals the presence of multiple nitrification inhibitors in biomass and root exudates of Thinopyrum intermedium
Source: Plant Environ Interact. 2024 Sep 27;5(5):e70012. doi: 10.1002/pei3.70012 (PMC11431351; doi:10.1002/pei3.70012)
Supplement: Supplementary file 2 — Data S2. [file PEI3-5-e70012-s001.pdf]

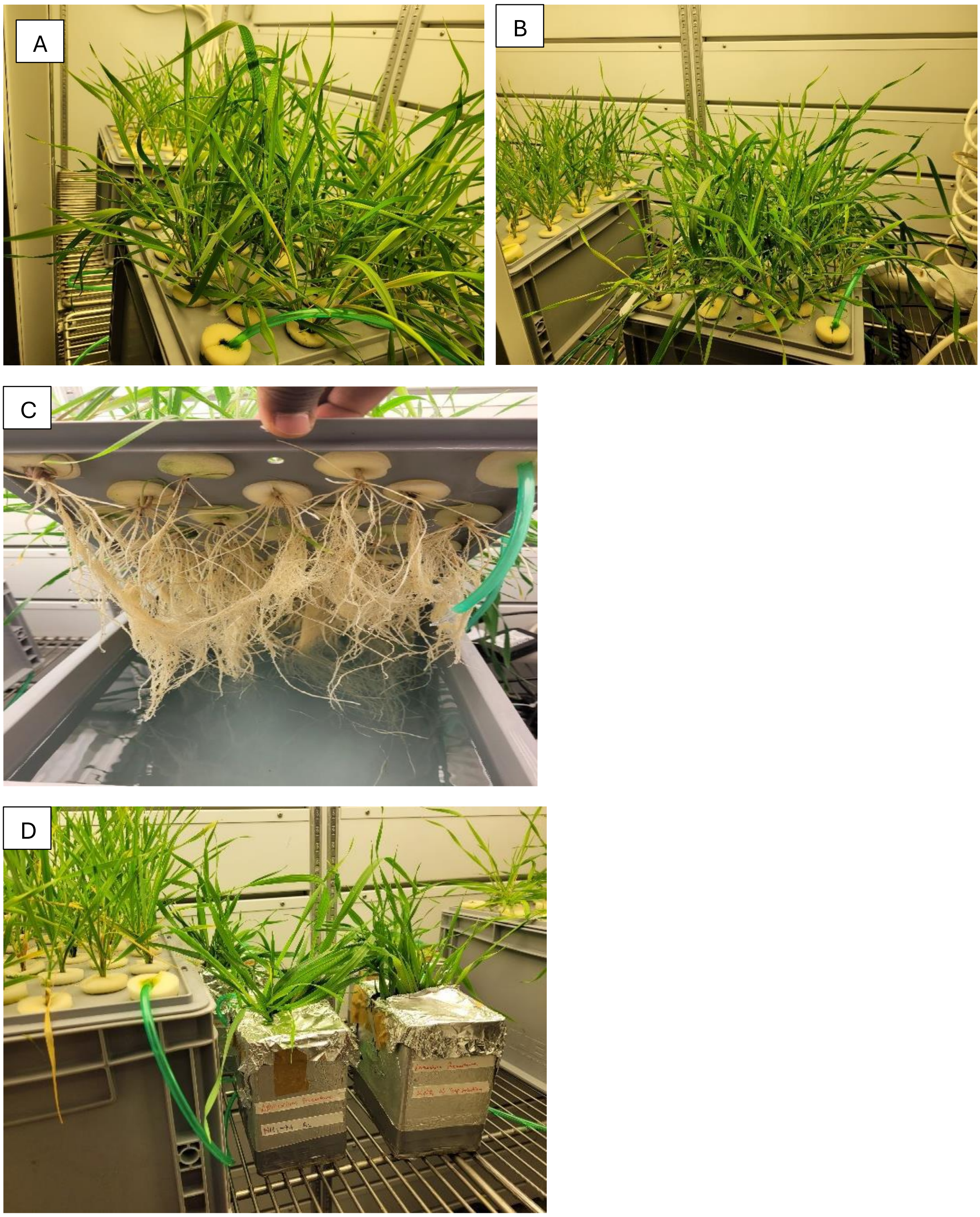

Notes

A and B show the tanks and growth state of crops during exudate collection. C shows the root development of Kernza as well as the aeration system used in the hydroponic system. D is the exudate collection in 1L glass boxes.
