# Supplementary material for: Metabolome fingerprinting reveals the presence of multiple nitrification inhibitors in biomass and root exudates of Thinopyrum intermedium
Source: Plant Environ Interact. 2024 Sep 27;5(5):e70012. doi: 10.1002/pei3.70012 (PMC11431351; doi:10.1002/pei3.70012)
Supplement: Supplementary file 3 — Data S3. [file PEI3-5-e70012-s007.pdf]

Metabolome fingerprinting reveals the presence of multiple nitrification inhibitors in biomass and root exudates of *Thinopyrum intermedium*

Sulemana Issifu[1], Prashamsha Acharya[1], Jochen Schöne[2], Jasmeet Kaur-Bhambra[3,4], Cecile Gubry-Rangin[3], Frank Rasche[1,5]

Analyte	RT	UV λmax
	(min)	(nm)
Protocatechuic Acid	2.45	258
Para Hydroxybenzoic Acid	3.85	254
Caffeic Acid	4.29	322
Para Coumaric Acid	5.52	309
Ferulic Acid	5.92	322
Benzoic Acid	6.4	229
Trans Cinnamic Acid	8.39	275
Quercetin	8.4	370
Gallic Acid	1.25	270
Umbelliferone	6.04	324
Vanillin	5.68	280
Vanillic Acid	4.27	291
Catechol	4.01	277
Coumarin	7.95	275
2,4DHBA	6.03	278
M3,4HPP	8.14	276
Syringic Acid	4.35	275
