# Supplementary material for: Metabolome fingerprinting reveals the presence of multiple nitrification inhibitors in biomass and root exudates of Thinopyrum intermedium
Source: Plant Environ Interact. 2024 Sep 27;5(5):e70012. doi: 10.1002/pei3.70012 (PMC11431351; doi:10.1002/pei3.70012)
Supplement: Supplementary file 4 — Data S4. [file PEI3-5-e70012-s003.pdf]

Preparation of samples for measurement:

- transfer an aliquot of 500 µL of the extracted sample into a new Eppendorf tube
- evaporate the ethyl acetate completely under nitrogen
- redissolve residue in 500 µL ACN/water (1/1)
- centrifuge 5 min, 12000 rpm
- transfer supernatant into a HPLC-vial

Measurement at the Thermo Fisher Accela HPLC-DAD

Column: YMC Triart C18/S-3µm/12nm, 100 x 3,0 mm

Eluents: 10 mM Formiate buffer pH 3,7 (B), ACN (C)

Injection volume 6 µL

Gradient:

	Time	A%	B%	C%	D%	µl/min	P2
0	0.00	0.0	90.0	10.0	0.0	500.0	
1	1.00	0.0	90.0	10.0	0.0	500.0	
2	15.00	0.0	10.0	90.0	0.0	500.0	
3	17.00	0.0	10.0	90.0	0.0	500.0	
4	17.10	0.0	90.0	10.0	0.0	500.0	
5	20.00	0.0	90.0	10.0	0.0	500.0	
6		100.0	0.0	0.0	0.0	500.0	

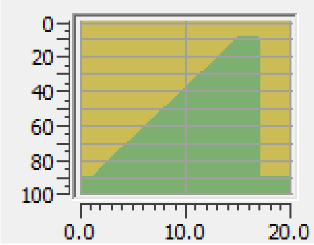

Solvent colors:

A B

C D

Type of view: Solvent Gradient

Analyte	RT	λ_{max} (nm)
**Protocatechuic Acid**	2.45	258
**Para Hydroxybenzoic Acid**	3.85	254
**Caffeic Acid**	4.29	322
**Para Coumaric Acid**	5.52	309
**Trans Ferulic Acid**	5.92	322
**Benzoic Acid**	6.40	229
**Trans Cinnamic Acid**	8.39	275
**Quercetin**	8.40	370
**Gallic Acid**	1.25	270
**Umbelliferone**	6.04	324
**Vanillin**	5.68	280
**Vanillic Acid**	4.27	291
**Anthraquinone**	13.48	252
**Catechol**	4.01	277
**Coumarin**	7.95	275
**24DHBA**	6.03	278
**M34HPP**	8.14	276
**Syringic Acid**	4.35	275

Standards concentrations: 0,1/1/5 mgL
