# Supplementary material for: Metabolome fingerprinting reveals the presence of multiple nitrification inhibitors in biomass and root exudates of Thinopyrum intermedium
Source: Plant Environ Interact. 2024 Sep 27;5(5):e70012. doi: 10.1002/pei3.70012 (PMC11431351; doi:10.1002/pei3.70012)
Supplement: Supplementary file 5 — Data S5. [file PEI3-5-e70012-s005.pdf]

Metabolome fingerprinting reveals the presence of multiple nitrification inhibitors in biomass and root exudates of *Thinopyrum intermedium*

Sulemana Issifu[1], Prashamsha Acharya[1], Jochen Schöne[2], Jasmeet Kaur-Bhambra[3,4], Cecile Gubry-Rangin[3], Frank Rasche[1,5]

Adjustment to ~ 8			
Original pH of media is 8			
		PH Buffer µL	
METABOLITE µM	PH EFFECT	NaCO3	10% HCl
Syringic acid 800	7.14	15	
Syringic acid 400	7.5	7	
Vanillin 200	7.9		
2,6 DHBA 1000	7	15	
2,6 DHBA 2000	6.77	26	
Phenylalanine 500	8		
Phenylalanine 1000	8		
Phenylalanine 200	8		
Methyl syringate 500	7.9		
Methyl syringate 1000	7.83		

Adjustment to ~ 7.5			
		PH Buffer µL	
METABOLITE µM	PH EFFECT	NaCO3	10% HCl
Control	8		1.7
Caffeic acid 500	7.47		
Caffeic acid 200	7.85		1.25
Phenylalanine	8		1.7
Vanillic acid + phenylalanine 200	7.8		1.3
Caffeic acid + phenylalanine 200	7.75		1.2
Vanillin + vanillic acid	7.64		1
Caffeic aid + vanillic acid	7.57		0.6
Vanillin + caffeic acid	7.54		0.5
Vanillic acid	7.87		1.25
