# Supplementary material for: Metabolome fingerprinting reveals the presence of multiple nitrification inhibitors in biomass and root exudates of Thinopyrum intermedium
Source: Plant Environ Interact. 2024 Sep 27;5(5):e70012. doi: 10.1002/pei3.70012 (PMC11431351; doi:10.1002/pei3.70012)
Supplement: Supplementary file 6 — Data S6. [file PEI3-5-e70012-s002.docx]

Metabolome fingerprinting reveals the presence of multiple nitrification inhibitors in biomass and root exudates of *Thinopyrum intermedium*

Sulemana Issifu^1^, Prashamsha Acharya^1^, Jochen Schöne^2^, Jasmeet Kaur-Bhambra^3,4^, Cecile Gubry-Rangin^3^, Frank Rasche^1,5^

^1^Institute of Agricultural Sciences in the Tropics (Hans-Ruthenberg-Institute), University of Hohenheim, Garbenstr. 13, 70599, Stuttgart, Germany.

^2^Institute of Phytomedicine, University of Hohenheim, Otto-Sander-Str. 5, 70599, Stuttgart, Germany

^3^School of Biological Sciences, Cruickshank Building, University of Aberdeen, St Machar Drive, Aberdeen, AB24 3UU, Scotland.

^4^Present address: Department of Plant and Environmental Sciences, University of Copenhagen, Thorvaldsensvej 40, 1871 Frederiksberg C, Denmark.

^5^Present address: International Institute of Tropical Agriculture, P.O. Box 30772-00100, Nairobi, Kenya.

Corresponding authors: Frank Rasche ([f.rasche@cgiar.org](mailto:f.rasche@cgiar.org)), Cecile Gubry-Rangin ([c.rangin@abdn.ac.uk](mailto:c.rangin@abdn.ac.uk))

Table 1. Putative and candidate metabolites annotated in root and leaf biomass of Kernza® and annual winter wheat.

|  | Kernza® | |  | Winter wheat | |
| --- | --- | --- | --- | --- | --- |
|  | Roots | Leaves |  | Roots | Leaves |
| Metabolites | Rt (min) | Rt (min) |  | Rt (min) | Rt (min) |
| Benzoic acid | 10.01 | 10.14 |  | 10.22 | 10.30 |
| Ferulic acid | 28.79 | 28.92 |  | 29.04 | 29.11 |
| *p* Hydroxybenzoic acid | 19.34 | 19.52 |  | 19.58 | 19.66 |
| Syringic acid | 25.20 | 25.35 |  | 25.49 | 25.49 |
| *p* Coumaric acid | 22.89 | 26.02 |  | 23.13 | 26.17 |
| Methyl *p* coumarate | 20.70 | n.f |  | n.f | n.f |
| Vanillic acid* | 22.40 | 22.56 |  | 22.64 | 22.72 |
| 2,6 dihydroxybenzoic acid* | n.f | 22.68 |  | n.f | n.f |
| Caffeic acid | n.f | 29.79 |  | n.f | n.f |
| Shikimic acid | n.f | 23.92 |  | n.f | n.f |
| alpha Linoleic acid | 30.97 | 31.13 |  | 31.44 | n.f |
| Protocatechuic acid | n.f | n.f |  | n.f | 23.95 |
| Oxalic acid | n.f | n.f |  | n.f | 12.46 |
| Phenylalanine | n.f | 19.53 |  | n.f | 19.76 |

* Metabolites noted as candidate compounds; n/f - not found; Rt – retention time in min.
